# Supplementary material for: A set of multi-entry identification keys to African frugivorous flies (Diptera, Tephritidae)
Source: Zookeys. 2014 Jul 24;(428):97–108. doi: 10.3897/zookeys.428.7366 (PMC4143993; doi:10.3897/zookeys.428.7366)
Supplement: Supplementary material 4 — Key to Capparimyia [file zookeys-428-097-s004.zip › SF4_ZooKeys_key to Capparimyia/lucid_player/help/help/restarting.htm]

Restarting a key


# Restarting a key

At the end of an identification click the Restart button  or use the *Restart*
menu item to reset the key for another identification. All features will
be unchecked and the *Features Chosen* and *Entities Discarded*
windows will be emptied.
  
 
  
If
the menu option *Reset Subsets*
on *Restart* is checked, the Player
will be restarted using the default subset defined by the key builder.
If this option is not checked, the key will restart using the current
feature and entity subsets.
